# Protective Effects of Kuding Tea (*Ilex kudingcha* C. J. *Tseng*) Polyphenols on UVB-Induced Skin Aging in SKH1 Hairless Mice

**DOI:** 10.3390/molecules24061016

**Published:** 2019-03-13

**Authors:** Ruokun Yi, Jing Zhang, Peng Sun, Yu Qian, Xin Zhao

**Affiliations:** 1 Chongqing Collaborative Innovation Center for Functional Food, Chongqing University of Education, Chongqing 400067, China; yirk@cque.edu.cn (R.Y.); sunpeng@foods.ac.cn (P.S.); qianyubaby@126.com (Y.Q.); 2 Chongqing Engineering Research Center of Functional Food, Chongqing University of Education, Chongqing 400067, China; 3 Chongqing Engineering Laboratory for Research and Development of Functional Food, Chongqing University of Education, Chongqing 400067, China; 4 Environment and Quality Inspection College, Chongqing Chemical Industry Vocational College, Chongqing 401228, China; zjinger0810@126.com

**Keywords:** Kuding tea, polyphenols, skin damage, SKH1 hairless mice, collagen

## Abstract

In this study, the protective effects of Kuding tea polyphenols (KTPs) on ultraviolet B (UVB)-induced skin injury of SKH1 hairless mice were studied. The ion precipitation method was used for extraction of polyphenols from Kuding tea. High-performance liquid chromatography showed that KTPs contains chlorogenic acid, cryptochlorogenic acid, isochlorogenic acid B, isochlorogenic acid A, and isochlorogenic acid C. SKH1 hairless mice were induced skin aging using 2.0 mW/s intensity of 90 mJ/cm^2^ UV light once a day for seven weeks. The 2.5% and 5% KTPs solution was smeared on 2 cm^2^ of back skin of skin aging mice twice a day. Mouse experiments showed that KTP strongly increased the serum levels of total superoxide dismutase (T-SOD) and catalase (CAT) and reduced those of malondialdehyde, interleukin 6 (IL-6), IL-1β, and tumor necrosis factor alpha (TNF-α) in mice with UVB-induced skin damage. KTP also increased the levels of type 1 collagen (Col I), hydroxyproline, and hyaluronic acid and reduced those of Col III and hydrogen peroxide in the damaged skin tissues of mice. Pathological observations of tissues stained with H & E, Masson’s trichrome, Verhoeff, and toluidine blue showed that KTPs could protect skin cells, collagen, and elastin and decrease the number of mast cells, thus inhibiting skin damage. Quantitative PCR and western blot assays showed that KTP upregulated the mRNA and protein expression of tissue inhibitor of metalloproteinase 1 (TIMP-1), TIMP-2, copper/zinc-SOD, manganese-SOD, CAT, and glutathione peroxidase and downregulated the expression of matrix metalloproteinase 2 (MMP-2) and MMP-9. In addition, the same concentration of KTP had stronger protective effects than vitamin C. The results of this study demonstrate that KTPs have good skin protective effects, as they are able to inhibit UVB-induced skin damage.

## 1. Introduction

Ultraviolet radiation, also known as ultraviolet (UV) light, is nonionizing radiation with wavelengths ranging between 100 and 400 nm. According to their wavelengths, UV can be divided into UVC (100–290 nm), UVB (290–320 nm), and UVA (320–400 nm) [1]. UVC is completely absorbed by the ozone layer and oxygen in the air. Proper exposure to UV can promote the synthesis of vitamin D3 in the skin, enhance excitability of the sympathetic adrenomedullary system, enhance immunity, and promote the secretion of some hormones [2]. However, excessive exposure to UV is linked to major health problems. Skin is the outermost layer of the body and its first line of defense [3]. Skin tissues are mainly composed of collagen fibers, elastic fibers, and proteoglycan-based matrix. Intrinsic aging is the natural process of skin aging due to internal factors, whereas extrinsic aging, often referred to as photoaging, is caused by external factors such as air pollution and UV radiation [4]. Currently, due to the increase in industrialization, the ozone layer has been severely damaged and there has also been a gradual increase in damage to the skin such as erythema, shriveling, roughness, lack of luster, and skin cancer [5,6]. UVB induces structural damage and content reduction of collagen fibers, denatures elastic fibers, and damages the extracellular matrix (ECM), leading to wrinkles, sagging, and other age-related symptoms, thereby damaging the skin and causing skin conditions [7,8].

The content of polyphenols in tea is generally 20–35%, the tea polyphenols include anthocyanins, flavonols, and phenolic acids [9]. Tea polyphenols are responsible for the color and flavor of tea and also have numerous health benefits [10]. Pharmacological studies have shown that tea polyphenols can reduce oxidative stress and cause vasodilation, regulate glucose levels, improve immune function, prevent UVB-induced oxidation of lipid peroxidation, and have anticarcinogenic properties [11,12]. Aging is caused by the accumulation of free radical damage over time; the main source of free radicals in the skin is UV light, which can cause homolysis of biomolecules and water molecules in the body and produce a large number of free radicals, thus damaging the skin [13]. Tea polyphenols can directly absorb UV light; at 290–320 nm, UVB has the most damaging effects on the skin. Tea polyphenols can absorb UV damage and scavenge free radicals, thereby preventing UV-induced damage to the skin [14]. Free radicals can directly attack biomolecules or radical-induced lipid oxidation products and further cross-link with biomolecules to damage the skin [8]. However, tea polyphenols can prevent this damage [15] by removing free radicals and reactive oxygen species (ROS) (O_2_^−^, OH, ^1^O_2_) [16,17,18]. They can also remove lipid radicals to block lipid peroxidation, and can inhibit cicatrice, pigmentation, skin inflammation, skin edema, and skin irritation [19].

Kuding tea is made from the evergreen tree leaves of *Ilex kudingcha* C. J. *Tseng*, which is common in southwest China. It is often used as a type of health drink and medicine, because according to Traditional Chinese Medicine, Kuding tea can relieve heat, quench thirst, and relieve coughing, meanwhile Kuding tea for external use can kill bacteria, reduce inflammation, and relieve itching [20]. The botanical classification and components of Kuding tea are different from those of green tea, so their biological activities also differ. Kuding tea and its active components have good antioxidant effects. Kuding tea is also rich in polyphenols, so it has anti-oxidant and anti-inflammatory properties as well [21]. Vitamin C is a very good antioxidant, which can eliminate the free radicals that lead to skin oxidation. These free radicals are also the culprits of skin aging. Therefore, skin care with vitamin C can slow down skin aging, prevent wrinkles and spots from occurring earlier, and achieve the effect of delaying aging [22]. Therefore, vitamin C was used as a positive control in this study.

The external application scope of Kuding tea is still relatively homemade, there is no research on its skin effect, and even less research on its polyphenols on skin effect. This study is the first to study the external effect and mechanism of Kuding tea polyphenols (KTPs) on skin. This study will open up a new application of Kudingcha polyphenols, so as to better exploit and utilize this natural plant resource. In this study, the components of KTPs were analyzed by HPLC and we established a mouse model of UVB irradiation-induced skin damage to observe the skin-protective effects of KTPs. The SKH1 hairless mice were induced skin aging using UV light, meanwhile KTPs solution was smeared on the back skin of skin aging mice before UV induction in the whole experiment. The serum levels of T-SOD, CAT, IL-6, IL-1β, TNF-α, and tissue levels of Col I, hydroxyproline, hyaluronic acid, Col III, and hydrogen peroxide were determined. The H & E, Masson’s trichrome, Verhoeff, and toluidine blue sections of skin tissue were also used for pathological observation. The mRNA and protein expression TIMP-1, TIMP-2, MMP-2, MMP-9, Cn/Zn-SOD, Mn-SOD, CAT, and GSH-Px were determined by qPCR and western blot assays. In addition, molecular biology methods were used to determine the mechanisms underlying the skin protective effects of KTPs and their constituents. The results showed that KTPs had good protective effects on the skin, and thus may be beneficial as a high-quality functional food resource. This study validates a new application method of KTPs, which will lay a theoretical foundation for further clinical research of Kuding Tea and provide a new idea for further utilization of Kuding tea resources.

## 2. Results

### 2.1. KTP Constituents

**Figure 1:**
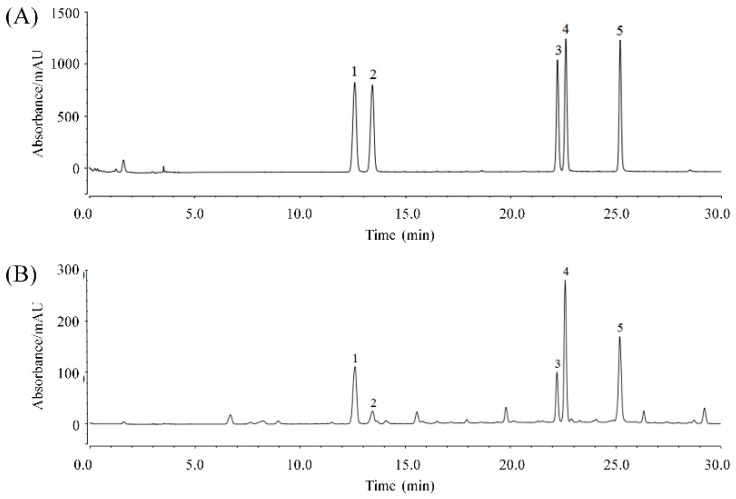
Determination of KTP constituents by HPLC. (**A**) Standard chromatograms; (**B**) KTP chromatograms (one sample). 1: chlorogenic acid; 2: cryptochlorogenic acid; 3: isochlorogenic acid B; 4: isochlorogenic acid A; and 5: isochlorogenic acid C.

As shown in Figure 1 and Table 1, the composition of the KTPs was chlorogenic acid, cryptochlorogenic acid, isochlorogenic acid A, isochlorogenic acid B, and isochlorogenic acid C at concentrations of 79.71 mg/g, 16.02 mg/g, 120.03 mg/g, 52.53 mg/g, and 96.42 mg/g, respectively.

### 2.2. Serum Levels of T-SOD, CAT, and MDA

The mice in healthy, UVB-treated, vitamin C (VC), low KTP concentration (LKTP), and high KTP concentration (HKTP) groups were treated as follows: the healthy group was not exposed to UV light; the UVB-treated group was exposed to UV light; the VC, LKTP, and HKTP groups were also exposed to UV light and smeared with 0.2 mL 5.0% VC, 2.5% KTPs, and 5.0% KTPs in solution on 2 cm^2^ of back skin. As shown in Table 2, serum levels of T-SOD and CAT in the healthy group were highest, but the MDA levels were lowest. After inducing skin injury by UVB, T-SOD, and CAT levels were decreased and MDA levels were increased. Smearing of VC and KTP in the skin also increased T-SOD and CAT levels and reduced MDA levels in mice, and the effects of 5% HKTP smearing were better than those of VC and LKTP treatment.

### 2.3. Serum Levels of IL-6, IL-1β, and TNF-α

As shown in Table 3, serum levels of IL-6, IL-1β, and TNF-α were lowest in mice in the healthy group and highest in the UVB-treated group. The IL-6, IL-1β, and TNF-α levels in mice of the HKTP group were lower than those of mice in the VC and LKTP groups; and IL-6, IL-1β, and TNF-α levels in mice of the LKTP group were only higher than those of mice in the UVB-treated group.

### 2.4. Col I, Col III, Hydroxyproline, and Hyaluronic Acid Contents in Skin Tissue

As shown in Table 4, the contents of Col I, hydroxyproline, and hyaluronic acid were highest in the healthy group mice, but Col III content was lowest in this group. UVB treatment reduced the Col I, hydroxyproline, and hyaluronic acid contents and increased Col III content. KTP and VC inhibited these changes after UVB treatment, and Col I, Col III, hydroxyproline, and hyaluronic acid contents of mice in the HKTP group were closest to those of the healthy group.

### 2.5. Total Protein and H_2_O_2_ of Skin Tissue

**Figure 2:**
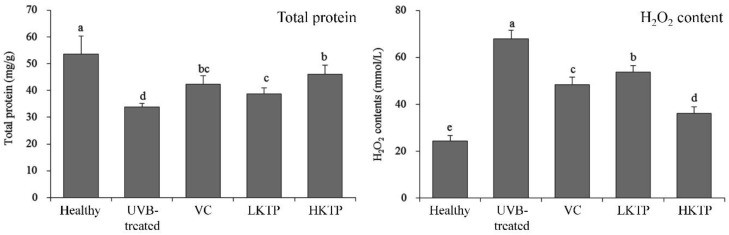
Total protein and H_2_O_2_ contents of skin tissue in SKH1 hairless mice with UVB-induced skin aging. Values presented are the means ± standard deviation (N = 10/group). ^a–d^ Mean values with different letters in the same bars are significantly different (*p* < 0.05) according to Duncan’s new MRT. VC: mice were smeared with 5.0% VC solution; LKTP: mice were smeared with 2.5% polyphenols of Kuding tea; and HKTP: mice were smeared with 5.0% polyphenols of Kuding tea.

As shown in Figure 2, the total protein content of mice in UVB-treated group was lowest, but this content of mice in healthy group was highest. After VC, LKTP, and HKTP treatment, the total protein contents of mice in VC, LKTP, and HKTP groups were raised compared to the UVB-treated group mice. The total protein content of mice in HKTP group was most close to the mice in healthy group. Meanwhile, the H_2_O_2_ content of mice in the UVB-treated group was highest, and HKTP, LKTP, and VC treatment reduced H_2_O_2_ content in mice with skin injury. The reducing effects of HKTP were stronger than those of LKTP and VC.

### 2.6. Histopathological Observations of Skin Tissue

**Figure 3:**
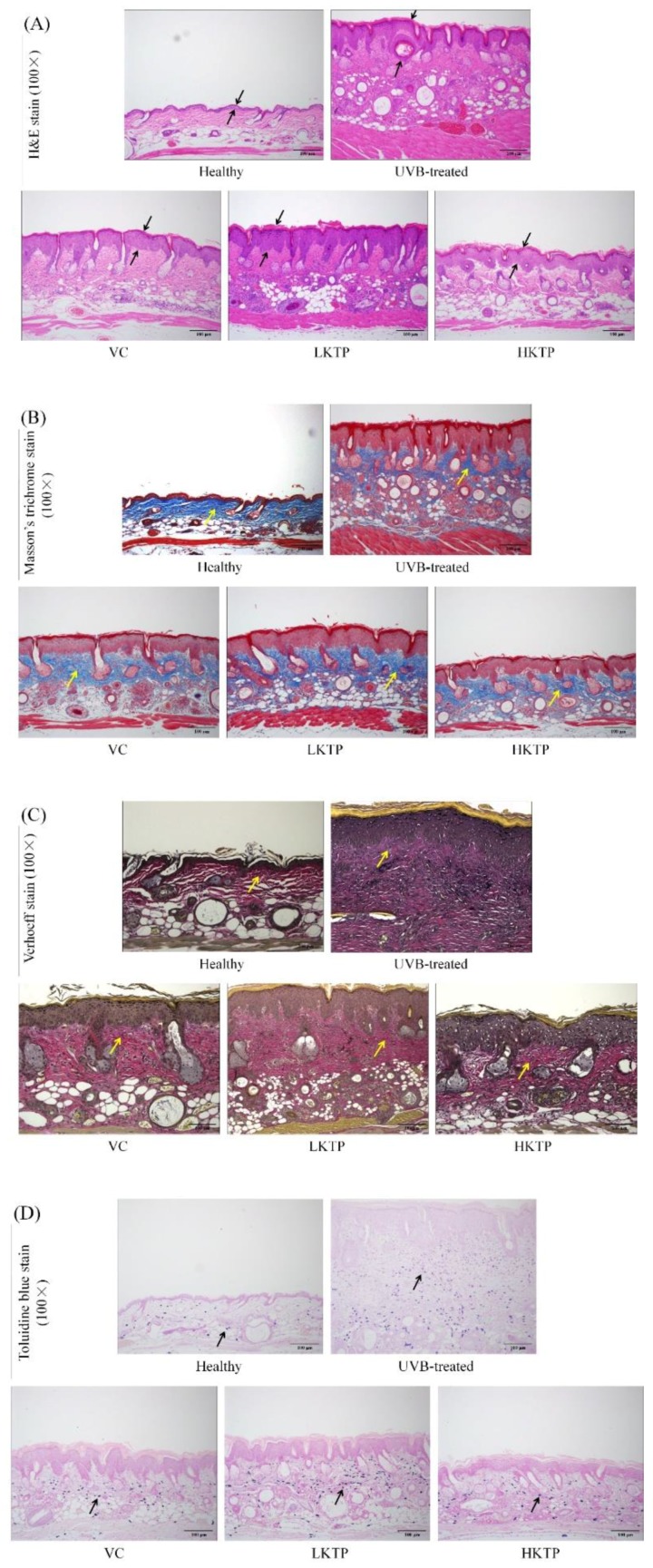
H & E (**A**), Masson’s trichrome (**B**), Verhoeff (**C**), and toluidine blue (**D**) staining of skin tissue in SKH1 hairless mice with UVB-induced skin aging. VC: mice were smeared with 5.0% VC solution; LKTP: mice were smeared with 2.5% polyphenols of Kuding tea; and HKTP: mice were smeared with 5.0% polyphenols of Kuding tea. Magnification 100×.

The representative tissue sections were shown in Figure 3, as a result of UVB stimulation, the epidermis and dermis of the skin swelled and thickened (Figure 3A). The blue part of the sliced tissue represented the collagen content, which is mainly found in the dermis of animals. The upper layer brown epidermis of the skin in the healthy group was thinner than that in the UVB-treated group, the skin epidermis of KTP treated skin damage mice became thinner. When collagen becomes cross-linked, the skin shows signs of aging such as wrinkles (Figure 3B). Compared with the dermis of healthy mice, the elastin of dermis of UVB-treated group was grossly overstained, and KTP could block this trend. Without elastin, the surface of skin loses elasticity and becomes stiff (Figure 3C). The black dots in the slice are mast cells, which are the source of histamine release in the irradiated skin and increase to inflammation. With the increase of mast cells, more inflammatory substances are secreted. The mast cells produced by UVB stimulation are associated with inflammation of the skin, counting on slices can be seen that there are 59 ± 4, 195 ± 13, 106 ± 8, 170 ± 11, and 72 ± 6 mast cells in healthy group, UVB-treated group, VC group, LKTP group, and HKTP group, respectively (Figure 3D). The mice in the UVB-treated group showed the above injuries in skin; treatment with HKTP reduced these conditions, and its effects were better than those of VC.

### 2.7. mRNA and Protein Expression of TIMP-1, TIMP-2, MMP-2, and MMP-9 in Skin Tissue

**Figure 4:**
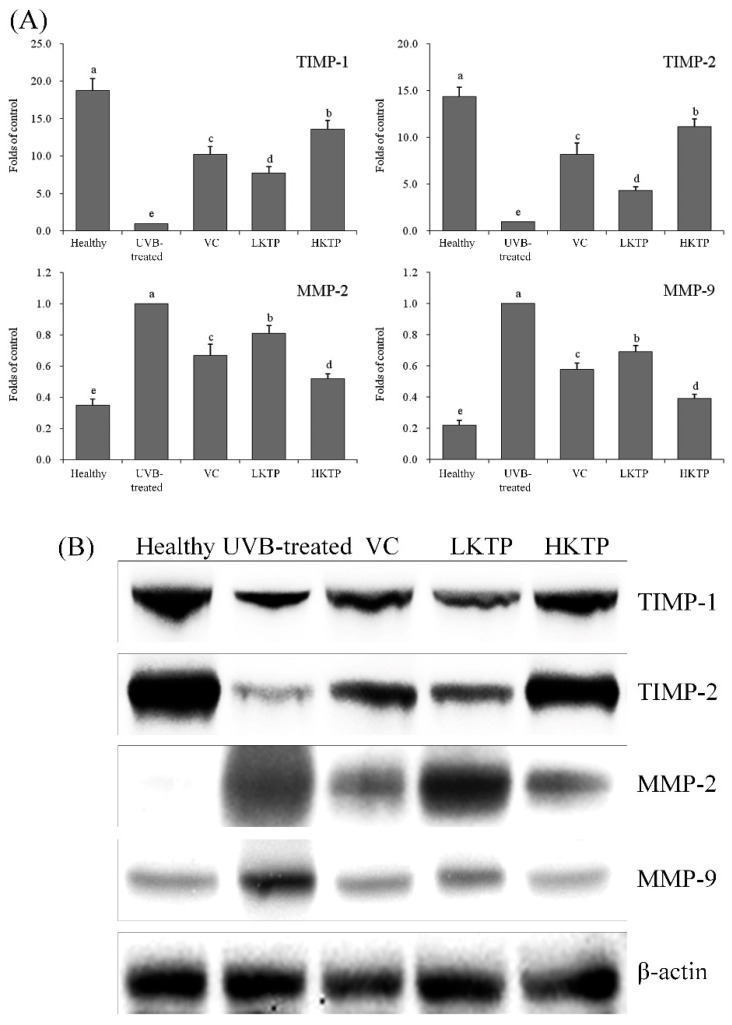
TIMP-1, TIMP-2, MMP-2, and MMP-9 mRNA (**A**) and protein (**B**) expression in skin tissue of SKH1 hairless mice with UVB-induced skin aging. Values presented are the means ± standard deviation (N = 10/group). ^a–e^ Mean values with different letters in the same bars are significantly different (*p* < 0.05) according to Duncan’s new MRT. VC: mice were smeared with 5.0% VC solution; LKTP: mice were smeared with 2.5% polyphenols of Kuding tea; and HKTP: mice were smeared with 5.0% polyphenols of Kuding tea.

As shown in Figure 4, the mRNA and protein expression of TIMP-1 and TIMP-2 was highest in the healthy group, and MMP-2 and MMP-9 expression was lowest in this group. UVB-treated group reduced TIMP-1 and TIMP-2 expression and increased MMP-2 and MMP-9 expression. PKT inhibited the effects of UVB, and the high concentration of PKT (HKTP) showed the best inhibitory effects. TIMP-1 and TIMP-2 expression in HKTP-treated mice was only weaker than expression in the healthy group, and MMP-2 and MMP-9 expression was only stronger than that of mice in the healthy group.

### 2.8. mRNA and Protein Expression of Cu/Zn-SOD, Mn-SOD, CAT, and GSH-Px in Skin Tissue

**Figure 5:**
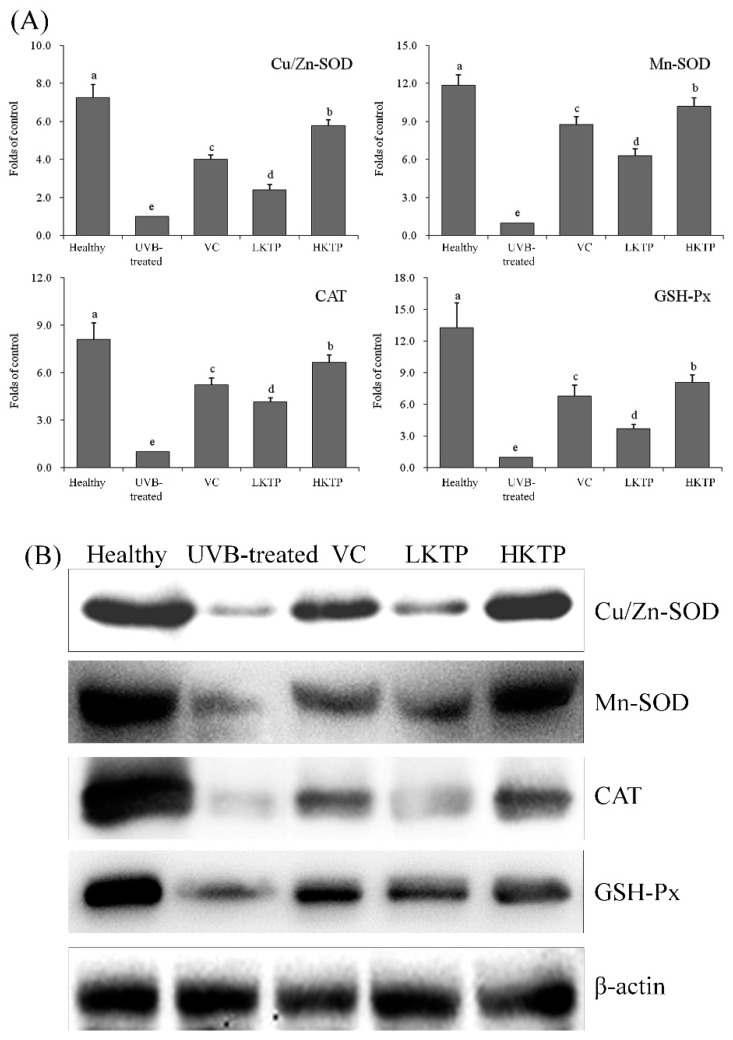
Cu/Zn-SOD, Mn-SOD, CAT, and GSH-Px mRNA (**A**) and protein (**B**) expression in skin tissue of SKH1 hairless mice with UVB-induced skin aging. Values presented are the means ± standard deviation (N = 10/group). ^a–e^ Mean values with different letters in the same bars are significantly different (*p* < 0.05) according to Duncan’s new MRT. VC: mice were smeared with 5.0% VC solution; LKTP: mice were smeared with 2.5% polyphenols of Kuding tea; and HKTP: mice were smeared with 5.0% polyphenols of Kuding tea.

As shown in Figure 5, the expression of Cu/Zn-SOD, Mn-SOD, CAT, and GSH-Px of mice in the healthy group were strongest. The expression of HKTP-treated mice was only weaker than that of mice in the healthy group, and VC-treated mice also showed stronger Cu/Zn-SOD, Mn-SOD, CAT, and GSH-Px expression than mice in the LKTP and UVB-treated groups.

## 3. Discussion

Of the UV rays, UVB is absorbed directly by DNA, which results in lesions and mutations, while ROS produced by UVB and UVA can indirectly damage the cell nucleus and mitochondrial DNA, leading to abnormal functions or apoptosis [23]. ROS is divided into two categories: free radicals (such as hyperoxygen free radicals and hydroxyl radicals) and non-free radical components (such as singlet oxygen and H_2_O_2_). UVB can produce ROS around skin cell membranes. Oxidation of ROS produced by UV irradiation can damage proteins and lipids, causing abnormalities of the corresponding function and structure [24]. Skin photoaging is a chronic injury of skin exposed to ultraviolet radiation. The penetrating power of UVB is strong, which can affect collagen and elastic fibers in dermal tissue, leading to skin photoaging. Active oxygen species are indirectly produced under the action of UVB, resulting in DNA oxidative damage and single and double strand breakage. The radiation energy of UVB is absorbed by proteins and DNA in cells, resulting in oxidative damage, which attacked DNA and triggered changes in MMPs expression. MMPs play a central role in the physiological mechanism of skin photoaging [23]. Therefore, this study focused on KTPs inhibiting the skin oxidative damage caused by UVB, thus preventing the light damage caused by MMPs to the skin.

SOD is an active substance derived from living organisms and it can eliminate harmful substances produced by organisms in the process of metabolism. Constant supplementation of SOD in the human body has special anti-aging effects [25]. Skin is the outermost layer of the body’s surface, so it is directly exposed to the radiation of sunlight and environmental pollution and is vulnerable to damage by oxygen-free radicals, which can cause phototoxicity, photoaging, and skin tumors. The skin cells have a series of complex antioxidant defenses, of which SOD is one of the most important enzymes. It can catalyze the dismutation reaction of superoxide anion-free radicals, balance oxygen-free radicals in the body, prevent the toxicity of oxygen, inhibit radiation damage, and prevent aging [26]. In mammalian tissues, there are three types of SOD isozymes: Cu/Zn-SOD, Mn-SOD, and extracellular-SOD. They have different expression and activity in different tissues and in different diseases, and all play independent roles in the body [27]. Cu/Zn-SOD is abundant in the cytoplasm, nucleus, and mitochondrial intermembrane space, where it reduces the concentration of O_2_^−^ in the cell, inhibits oxidative damage, and delays aging [28]. Mn-SOD is a mitochondrial enzyme that is localized in the mitochondrial matrix, where it can eliminate O_2_^−^ produced by respiratory chain-related reactions and prevent lipid peroxidation damage. Although there are fewer mitochondria in the skin, Mn-SOD still has functions in this organ [29]. In this study, KPT increased SOD activities in mice with skin damage for skin protection. Because it is a strong oxidizing agent, H_2_O_2_ can cause lipid peroxidation and oxidative damage to skin. CAT is an enzyme scavenger that can decompose H_2_O_2_ into molecular oxygen and water and remove hydrogen peroxide from the body, thus preventing cells from being poisoned by H_2_O_2_; thus, it is a key enzyme in the body’s defense system [30]. Widely existing in the human body, GSH-Px is an important enzyme that catalyzes the decomposition of H_2_O_2_ and decreases free radicals, thereby protecting the structure and function of the cell membrane and preventing oxidative damage caused by UVB irradiation [31]. UVB stimulates the skin to produce free radicals, which causes lipid peroxidation of which the end product is MDA. MDA causes crosslinking polymerization of macromolecules such as protein and nucleic acid and is cytotoxic. Thus, inhibiting MDA activity can protect the skin from damage [32]. In this study, KTPs increased CAT activity and reduced MDA activity, thereby decreasing UVB-induced damage.

Keratinocytes are the main targets of UV radiation. By releasing pre-inflammatory cytokines such as IL-1, IL-6, IL-8, IL-10, and TNF-α, keratinocytes play central roles in UV light damage [33]. UV radiation induces the formation of networks of IL-1α, IL-1β, and IL-6, and the inter-related secretion cycle induces collagenase/MMP-1 [34,35]. Through self-secretion, IL-6 induces MMPs. The expression of MMPs promotes degradation of the ECM, and MMPs also play a role in skin cancer induced by UV radiation [36]. Through cell surface receptors, IL-1β and TNF-α not only promote the transmission of NF-κB signals, but also activate the transcription of activator protein 1-regulated genes by activating the mitogen-activated protein kinase signaling pathway, inducing the expression of MMPs [37]. Upon UVB radiation of the skin, keratinocytes can indirectly cause skin photoaging by causing the secretion of IL-1β and IL-6. IL-1α, IL-1β, and TNF-α can increase the synthesis and release of IL-6 in keratinocytes [38]. Thus, the fact that KTPs decreased the levels of IL-6, IL-1β, and TNF-α demonstrates its skin-protective properties.

ECM components are closely related to the mechanical properties of the skin, and the stable structure and arrangement of ECM components are the basis of good skin elasticity. Collagen accounts for 75% of skin protein and serves to maintain the elasticity and moisture of the skin; thus, collagen content in skin is an important indicator of skin aging [39]. There are different types of collagen in skin, of which Col I and Col III are mainly associated with elasticity. In the process of skin aging, the content of Col I decreases whereas that of Col III increases, so adjusting the ratio between Col I and Col III has anti-aging effects [40]. Hydroxyproline is a non-essential amino acid comprising about 13% of total collagen and can directly reflect the content variation of collagen fibers in dermis; thus, it is used as a sensitive indicator of the degree of skin aging [41]. Hyaluronic acid not only plays an important role in maintaining skin moisture and structure, but also promotes skin regeneration, enhances skin elasticity, and degrades free radicals in the skin [42]. MMPs can degrade ECM components, decrease the content of collagen I, and decrease the ratio of collagen I/collagen III. These are the obvious manifestations of skin damage caused by ultraviolet radiation [43]. In this study, KTP also increased levels of Col I, hydroxyproline, hyaluronic acid, and reduced Col III, showing its role in protecting against skin damage.

The main components of the skin are proteins, fat, carbohydrates, water, and electrolytes. Proteins comprise the epidermis and dermis of the skin and are the main components of collagen fibers, which serve to maintain the elasticity of the skin. H_2_O_2_ can damage proteins and aggravate skin oxidative damage [43]. We found that KTPs could also inhibit UVB-induced skin damage by reducing H_2_O_2_ levels in skin tissue.

MMPs participate in the degradation of many ECM components. Because of its active expression in the wound repair process, MMPs have response to a living injury. Studies have shown that MMP-2 and MMP-9, also known as gelatinase, not only continuously degrade interstitial fiber collagen, but also play important roles in the reconstruction of ECM collagen [44]. Although they damage the ECM close to the cell surface and the basement membrane, capillary endogenesis and neovascularization promote angiogenesis and migration of vascular smooth muscle, thereby promoting the infiltration and metastasis of tumors [45]. In the wound repair process, MMP-2 and MMP-9 are mainly involved in dissolution of the basement membrane, formation of blood vessels, and the removal of necrotic tissues [46].

TIMPs irreversibly combine with MMPs to specifically inhibit their activity, thus preventing the infiltration and metastasis of tumors. TIMP-2 and TIMP-1 can non-covalently bind with MMP-2 and MMP-9, respectively, to prevent direct damage to the ECM caused by the transformation process from proenzyme to active enzyme [47]. As the expression of TIMP-1 and TIMP-2 decreases, the balance between MMPs and TIMPs is disrupted, which weakens the inhibitory effects of TIMPs on MMPs and causes the secretion of a large amount of MMPs, resulting in skin damage and even pathological changes [48]. In this study, KTP increased TIMP-1 and TIMP-2 expression and decreased MMP-2 and MMP-9 expression in mice with skin damage.

Collagen is the main component of biomacromolecules and animal connective tissues. Elastin is the main component of elastic fibers. Elastic fibers mainly exist in ligaments and vasculature [49]. Elastic fibers and collagen fibers are the basis for tissue flexibility and resilience, making them very important components of the skin [50]. Long-term UVB radiation causes inflammation of the skin and damages its internal microstructure, resulting in wrinkles, fine lines, and cutis laxa. Labrocytes widely exist around microvessels under the skin and visceral mucosa, and hyperplasia of labrocytes can occur in skin diseases including measles [51]. KTPs could keep the collagen and elastin in skin damage mice, KTPs also played an important role in the inhibition of skin damage.

In this study, the KTPs were analyzed by HPLC. It was found that there were five main compounds in KTPs. Because peak 2 (cryptochlorogenic acid) had a small shoulder, there was a certain error in calculating the peak area by automatic integration. In this study, the peak area was calculated by manual integration, and the content was calculated. Kuding tea is a drink that also has medicinal functions, as in traditional Chinese medicine, Kuding tea is used to treat headaches, toothaches, and dysentery. It has also been shown to play a role in alleviating hypertension, obesity, stomatitis, pharyngitis, acute gastritis, abdominal pain, fever, gas, and constipation. However, the specific mechanisms underlying its effects remain unknown. In this study, the polyphenols of Kuding tea were extracted and separated, and their components were analyzed. Previous studies have evaluated the individual effects of these active components. Chlorogenic acid contains a special ingredient that can promote the synthesis and decomposition of gelatin in the skin and muscle. It also has clear scavenging effects of free radicals both in vivo and in vitro [52]. At the same time, chlorogenic acid has inhibitory effects on the production of hyaluronic acid and glucose-6-phosphatase, resulting in wound healing, skin health and hydration, and prevention of inflammation [53]. Cryptochlorogenic acid is a component of traditional Chinese medicine that often works in cooperation with other active ingredients to produce anti-inflammatory and anti-aging effects [54,55]. Isochlorogenic acid A has strong antioxidant effects and was shown to effectively remove free radicals both in vitro and in zebrafish [56]. It has been shown that both chlorogenic acid and isochlorogenic acid A have bacteriostasis, which may be one mechanism underlying their skin-protective effects [57]. Isochlorogenic acid B and isochlorogenic acid C also have some functional effects, such as anti-hepatic and antioxidant effects [58,59]. KTP contains these active ingredients, which may explain its effects in anti-oxidation and inhibition of UVB-induced skin damage.

## 4. Materials and Methods

### 4.1. KTP Extraction

To extract polyphenols from Kuding tea in one batch, 500 g Kuding tea (Wuhu City Huichapin Tea Co. Ltd., Wuhu, Anhui, China) was dried and crushed, and 5 L ethanol solution (45%, *v*/*v*) was added to the Kuding tea powder (90 °C, 30 min). For extraction, the pH was adjusted to 6.0 and 800 mL mixed precipitant was added (100 g aluminum trichloride and 200 g zinc chloride were dissolved in 2.7 L water). After centrifugation at 1409× *g* for 10 min, the precipitate was separated. Then 1 L hydrogen chloride (12%, *v*/*v*) was added to the precipitate, and the supernatant solution was separated. Finally, 1 L ethyl acetate was added to the supernatant solution and extracted twice, after which the two extracts were mixed together and dried by rotary evaporation (R-1001-VN; Zhengzhou Greatwall Scientific Industrial and Trade Co., Ltd., Zhengzhou, Henan, China). In this way, the polyphenols from Kuding tea were obtained.

### 4.2. High-Performance Liquid Chromatography

The constituents of the KTPs were analyzed by high-performance liquid chromatography (HPLC) (Ultimate3000; Thermo Fisher Scientific, Inc., Waltham, MA, USA). 20 mg of chlorogenic acid, cryptochlorogenic acid, isochlorogenic acid A, isochlorogenic acid B, and isochlorogenic acid C standard weighed precisely and put into different 20 mL volumetric bottles respectively. 50% methanol (*v*/*v*) was added to dissolve the standard and volume to the scale. This is the reference reserve solution. Then five reference substance reserve solutions of 1 mL were separately taken out and put into 10 mL volumetric bottles. 50% methanol was used to constant volume to scale, that was the standard solution. The chromatographic conditions were as follows: AcclaimTM120 C18 column (4.6 × 150 mm, 5 μm); mobile phase: methanol (mobile phase A)-0.5% glacial acetic acid (mobile phase B) (Table 5); detection wavelength: 328 nm; column temperature: 35 °C; flow rate: 1 mL/min; injection volume: 20 μL; retention time of five main chromatographic peaks was 12.623, 13.453, 22.220, 22.613, 25.210 min. The extract of KTPs was filtered using a 0.45 μm microporous filter membrane, and 20 μL was used for HPLC.

### 4.3. Skin Injury Induced by UV Radiation

Six-week-old female SKH1 hairless mice (n = 50) were purchased from the Animal Center of Shanghai Public Health Clinical Center (Shanghai, China). The mice were divided into five groups: healthy, UVB-treated, vitamin C (VC), low KTP concentration (LKTP), and high KTP concentration (HKTP) groups. There were 10 mice in each group, during the experiment, all mice were allowed to receive standard food and water ad libitum. Mice were fed in a standard environment (25 ± 2 °C temperature, 50 ± 5% relative humidity, 12 h light/dark cycle) for one week. Then the mice were treated as follows: the healthy group was only fed food and water for seven weeks; the UVB-treated group was exposed to UV light using a UV radiation device (UV lamp FS40; Candela Corp., Santa Ana, CA, USA) for seven weeks, the mice were fixed at the bottom of the wooden box. UVB light (2.0 mW/s intensity) was installed at the top of the wooden box, 30 cm away (90 mJ/cm^2^) from the bottom of the box. The mice were irradiated by UVB light for 3 min at 9:00 a.m. every day. The mice in VC, LKTP, and HKTP groups were also exposed to UV light as the mice in UVB-treated group, each mouse was evenly smeared with 0.2 mL 5.0% VC, 2.5% KTPs, and 5.0% KTPs in solution on 2 cm^2^ of back skin using a medical sterile cotton swab once before and after irradiation every day. The mice were sacrificed using carbon dioxide after 7 weeks, and the skin tissues and plasma were collected for further determination. The experimental protocol was approved by the Animal Ethics Committee of Chongqing Medical University (Laboratory animal using license No. SYXK (Yu) 2018-0003, Chongqing, China).

### 4.4. Determination of Oxidation-Related Serum Levels

The collected blood was kept at 37 °C for 1 h, then the blood was centrifuged (1409× *g*, 10 min, 4 °C) and the serum was prepared. The serum levels of total superoxide dismutase (T-SOD; No. A001-1-1), catalase (CAT; No. A007-2) and malondialdehyde (MDA; No. A003-1) were determined using kits (Nanjing Jiancheng Bioengineering Institute, Nanjing, Jiangsu, China).

### 4.5. Determination of Serum Cytokine Levels

Serum levels of cytokines interleukin 6 (IL-6; no. ab46100), IL-1β (no. ab100704), and tumor necrosis factor alpha (TNF-α; no. ab100747) were determined with enzyme-linked immunoassay (ELISA) kits (Abcam, Cambridge, MA, USA).

### 4.6. Pathological Observations of Skin Tissue

The collected skin tissues were cleaned in saline, fixed in buffered formalin (10%, *v*/*v*) for 24 h, cut into two longitudinal halves, and embedded into paraffin. Paraffin sections 4 μm thick were stained with hematoxylin and eosin (H & E), Masson’s trichrome, Verhoeff, and toluidine blue prior to microscopy (BX43; Olympus, Tokyo, Japan).

### 4.7. Determination of Protein Levels in Skin Tissue

Saline was added to skin tissue, and the tissue was homogenized (Bioprep-24 biological sample homogenizer, All For Life Science, Hangzhou, Zhejiang, China) at 3578× *g* for 10 s. The skin tissue levels of type 1 collagen (Col I; no. H142), Col III (no. H144), hydroxyproline (no. A030-2), hyaluronic acid (no. H141), total protein (no. A045-3), and hydrogen peroxide (H_2_O_2_) (no. A064) were determined by the kits (Nanjing Jiancheng Bioengineering Institute).

### 4.8. Quantitative Polymerase Chain Reaction

The skin tissues of mice were collected, and the tissues were washed with normal saline. Total RNA was extracted with Trizol reagent (Invitrogen, Carlsbad, CA, USA) according to the manufacturer’s instructions. Then 1 μg extracted RNA was mixed with a cocktail reagent containing 1 μL oligodT_18_, 1 μL RNase, 1 μL dNTP, 1 μL murine leukemia virus enzyme, and 10 μL 5× buffer to synthesize cDNA using the conditions of 37 °C for 120 min; 99 °C for 4 min, and 4 °C for 3 min. Then 2 μL cDNA was mixed with 2 μL of 10 μM primer, 10 μL 2×SYBR Premix Ex Taq II, 0.4 μL 50 × ROX reference Dye, and 5.6 μL ddH_2_O. Quantitative PCR (qPCR) was performed in an automatic thermocycler (StepOnePlus Real-Time PCR System; Thermo Fisher Scientific) for 40 cycles at 94 °C for 30 s, 58 °C for 30 s, and 72 °C for 50 s, followed by a 10 min at 75 °C. The relative mRNA transcription levels (Table 6) were calculated according to the 2^−ΔΔCr^ formula [26].

### 4.9. Western Blot Analysis

The skin tissues of mice were collected, and total protein from skin tissues was extracted using radioimmunoprecipitation assay buffer (Easybio, Beijing, China). Then the extracted protein was centrifuged at 13,000× *g* for 30 min at 4 °C, followed by denaturation by boiling at 98 °C. The concentrations of the extracted protein were determined using the Quartzy Quick Start Bradford Protein Assay Kit (Bio-Rad Laboratories, Hercules, CA, USA). Then 30 µg protein was separated on 10–12% SDS-PAGE gels (Schleicher and Schuell, Keene, NH, USA) for 4 h and transferred to nitrocellulose membranes. Membranes were sealed for 3 h in sealing fluid, washed three times with phosphate-buffered saline (PBS) containing 0.05% Tween 20 (PBS-T), and incubated with primary antibodies for 2 h against tissue inhibitor of metalloproteinase 1 (TIMP-1) (no. MA1-773; Thermo Fisher Scientific), TIMP-2 (no. MA1-774; Thermo Fisher Scientific), MMP-2 (no. MA5-13590; Thermo Fisher Scientific), MMP-9 (no. MA5-15886; Thermo Fisher Scientific) copper/zinc (Cu/Zn)-SOD (no. MA1-105, Thermo Fisher Scientific), manganese (Mn)-SOD (no. LF-MA0030; Thermo Fisher Scientific), CAT (no. LF-PA0060; Thermo Fisher Scientific), glutathione peroxidase (GSH-Px) (no. PA5-40504; Thermo Fisher Scientific), and β-actin (no. MA5-15739; Thermo Fisher Scientific). Membranes were washed with PBS-T and incubated with horseradish peroxidase (HRP)-conjugated goat anti-mouse secondary antibody (no. A21241, Thermo Fisher Scientific) for 1 h at 25 °C. Following three washes with PBS-T, the membranes were incubated with enhanced chemiluminescence HRP substrates (Millipore, Billerica, MA, USA) and proteins were visualized using the Tanon Luminescent Imaging Workstation (6200; Shanghai Tanon Technology Co., Ltd., Shanghai, China) [26].

### 4.10. Statistical Analysis

The data are presented as the mean ± standard deviation. Differences between the mean values for mice in each group were assessed by one-way analysis of variance with the Duncan’s multiple range test. *p* < 0.05 was considered statistically significant. The SAS version 9.1 statistical software package (SAS Institute Inc., Cary, NC, USA) was used for the analyses.

## 5. Conclusions

In this study, the KTP prevented the effects of UVB-induced skin injury in SKH1 hairless mice. KTP could increase the serum levels of T-SOD and CAT and reduce the levels of MDA. It also could reduce the serum levels of IL-6, IL-1β, and TNF-α in these mice. Histological observation of skin tissues showed that KTP could increase the total protein levels of Col I, hydroxyproline, and hyaluronic acid, and decrease Col III and H_2_O_2_ contents compared to untreated mice (UVB-treated group). By qPCR and western blot analyses, KTP was shown to upregulate the mRNA and protein expression of TIMP-1, TIMP-2, Cu/Zn-SOD, Mn-SOD, CAT, and GSH-Px mRNA and downregulate MMP-2 and MMP-9 expression. These results demonstrated that KTP could protect the skin from UVB-induced skin damage in mice, and the effects were stronger than those of VC at the same concentration. The effects of KTPs may be from the synergistic effects of its active components, namely chlorogenic acid, cryptochlorogenic acid, isochlorogenic acid B, isochlorogenic acid A, and isochlorogenic acid C. Thus, as a functional component extracted from foods, KTP has good protective effects on the skin in mice. The results of this study expand the application of KTPs, which is conducive to the further application of traditional foods. Clinical experiments are needed in future studies to demonstrate the application and effects of KTP in human skin.

## Figures and Tables

**Table 1:** Determination of KTP constituents by HPLC (one sample).

Peak No.	Constituent	Retention Time (min)	Content (mg/g)	Theoretical Plate Number	Resolution
1	Chlorogenic acid	12.623	79.71	22586	2.56
2	Cryptochlorogenic acid	13.453	16.02	29063	34.37
3	Isochlorogenic acid B	22.220	52.53	206572	1.88
4	Isochlorogenic acid A	22.613	120.03	164025	11.05
5	Isochlorogenic acid C	25.210	96.42	165480	5.33

**Table 2:** Serum levels of T-SOD, CAT, and MDA in SKH1 hairless mice with UVB-induced skin aging.

Group	T-SOD (U/mL)	CAT (U/mL)	MDA (nmol/mL)
Healthy	107.36 ± 5.71 ^a^	13.36 ± 0.31 ^a^	10.62 ± 0.25 ^e^
UVB-treated	68.71 ± 3.44 ^e^	7.85 ± 0.25 ^e^	21.03 ± 0.65 ^a^
VC	84.34 ± 3.02 ^c^	9.76 ± 0.25 ^c^	15.21 ± 0.42 ^c^
LKTP	77.62 ± 2.61 ^d^	8.99 ± 0.18 ^d^	18.33 ± 0.61 ^b^
HKTP	93.56 ± 3.26 ^b^	11.89 ± 0.32 ^b^	13.29 ± 0.46 ^d^

Values presented are the means ± standard deviation (N = 10/group). ^a–e^ Mean values with different letters in the same column are significantly different (*p* < 0.05) according to Duncan’s new MRT. VC: mice were smeared with 5.0% VC solution; LKTP: mice were smeared with 2.5% polyphenols of Kuding tea; and HKTP: mice were smeared with 5.0% polyphenols of Kuding tea.

**Table 3:** Serum levels of cytokines IL-6, IL-1β, and TNF-α in SKH1 hairless mice with UVB-induced skin aging.

Group	IL-6 (pg/mL)	IL-1β (pg/mL)	TNF-α (pg/mL)
Healthy	10.77 ± 0.35 ^e^	37.52 ± 1.59 ^e^	44.36 ± 3.78 ^e^
UVB-treated	18.33 ± 0.61 ^a^	64.39 ± 3.15 ^a^	71.26 ± 4.58 ^a^
VC	14.37 ± 0.36 ^c^	50.32 ± 2.87 ^c^	52.34 ± 2.30 ^c^
LKTP	16.11 ± 0.28 ^b^	57.17 ± 1.88 ^b^	64.59 ± 3.51 ^b^
HKTP	12.58 ± 0.32 ^d^	44.02 ± 2.03 ^d^	47.33 ± 1.89 ^d^

Values presented are the means ± standard deviation (N = 10/group). ^a–e^ Mean values with different letters in the same column are significantly different (*p* < 0.05) according to Duncan’s new MRT. VC: mice were smeared with 5.0% VC solution; LKTP: mice were smeared with 2.5% polyphenols of Kuding tea; and HKTP: mice were smeared with 5.0% polyphenols of Kuding tea.

**Table 4:** Col I, Col III, hydroxyproline, and hyaluronic acid in the skin tissue of SKH1 hairless mice with UVB-induced skin aging.

Group	Col I (mg/g)	Col III (mg/g)	Hydroxyproline (mg/g)	Hyaluronic Acid (μg/g)
Healthy	21.33 ± 2.77 ^a^	5.42 ± 0.82 ^e^	81.36 ± 5.69 ^a^	536.72 ± 27.86 ^a^
UVB-treated	7.35 ± 1.21 ^e^	19.37 ± 1.67 ^a^	32.02 ± 3.35 ^e^	148.36 ± 13.53 ^e^
VC	12.36 ± 1.58 ^c^	10.35 ± 1.28 ^c^	52.36 ± 2.92 ^c^	312.05 ± 22.38 ^c^
LKTP	9.78 ± 0.68 ^d^	13.14 ± 2.10 ^b^	46.20 ± 2.43 ^d^	277.06 ± 18.75 ^d^
HKTP	16.39 ± 2.28 ^b^	7.79 ± 0.63 ^d^	66.71 ± 4.86 ^b^	441.25 ± 25.16 ^b^

Values presented are the means ± standard deviation (N = 10/group). ^a–e^ Mean values with different letters in the same column are significantly different (*p* < 0.05) according to Duncan’s new MRT. VC: mice were smeared with 5.0% VC solution; LKTP: mice were smeared with 2.5% polyphenols of Kuding tea; and HKTP: mice were smeared with 5.0% polyphenols of Kuding tea.

**Table 5:** Proportion of gradient elution mobile phase

Time/min	Mobile Phase A%	Mobile Phase B%
0	12	88
0–20	12→44	88→56
20–23	44→50	56→50
23–30	50→55	50→45

**Table 6:** qPCR assay sequences.

	Forward Sequence	Reverse Sequence
TIMP-1	5′-ATGCGGCCGCATGATGGCCCCCTTTGCATC-3′	5′-ATCCCGGGTCATCGGGCCCCAAGGGATC-3′
TIMP-2	5′-GTAGTGATCAGGGCCAAAG-3′	5′-TTCTCTGTGACCCAGTCCAT-3′
MMP-2	5′-ACCGAGGACTATGACCGGGATAA-3′	5′-GTCCTCATACTTGTTGCCCAGGA-3′
MMP-9	5′-GCCCTGGAACTCACACGACA-3′	5′-TTGGAAATCCACACGCCAGAAG-3
iNOS	5′-GTTCTCAGCCCAACAATACAAGA-3′	5′-GTGGACGGGTCGATGTCAC-3
Cu/Zn-SOD	5′-AACCAGTTGTGTTGTCAGGAC-3′	5′-CCACCATGTTTCTTAGAGTGAGG-3′
Mn-SOD	5′-CAGACCTGCCTTACGACTATGG-3′	5′-CTCGGTGGCGTTGAGATTGTT-3′
CAT	5′-GGAGGCGGGAACCCAATAG-3′	5′-GTGTGCCATCTCGTCAGTGAA-3
GSH-Px	5′-CCACCGTGTATGCCTTCTCC-3′	5′-AGAGAGACGCGACATTCTCAAT-3′
GAPDH	5′-AGGTCGGTGTGAACGGATTTG-3′	5′-GGGGTCGTTGATGGCAACA-3′
